# Glove-Induced Hand Dermatitis: A Study in Healthcare Workers during COVID-19 Pandemic in Indonesia

**DOI:** 10.1155/2023/6600382

**Published:** 2023-08-02

**Authors:** Cita Rosita Sigit Prakoeswa, Damayanti, Sylvia Anggraeni, Menul Ayu Umborowati, Fajar Waskito, Niken Indrastuti, Sri Awalia Febriana, Agnes Rosarina Prita Sari, Kristo Alberto Nababan, Cut Putri Hazlianda, Nopriyati, Windy Keumala Budianti, Miranti Pangastuti, Faridha Ilyas, Agnes Kartini, Nurwestu Rusetiyanti, Ika Anggraini, Idrianti Idrus, Herwinda Brahmanti, Gardenia Akhyar

**Affiliations:** ^1^Department of Dermatology and Venereology, Faculty of Medicine Universitas Airlangga/Dr. Soetomo General Academic Hospital, Surabaya, Indonesia; ^2^Department of Dermatology and Venereology, Dr. Sardjito General Hospital, Yogyakarta, Indonesia; ^3^Department of Dermatology and Venereology, H. Adam Malik General Hospital, Medan, Indonesia; ^4^Department of Dermatology and Venereology, Sumatera Utara University Hospital, Medan, Indonesia; ^5^Department of Dermatology and Venereology, Dr. M. Hoesin General Hospital, Palembang, Indonesia; ^6^Department of Dermatology and Venereology, Dr. Cipto Mangunkusumo National Central Public Hospital, Jakarta, Indonesia; ^7^Department of Dermatology and Venereology, Dr. Hasan Sadikin General Hospital, Bandung, Indonesia; ^8^Department of Dermatology and Venereology, Dr. Wahidin Sudirohusodo General Hospital, Makassar, Indonesia; ^9^Department of Dermatology and Venereology, Abdoel Wahab Sjahranie Regional General Hospital, Samarinda, Indonesia; ^10^Department of Dermatology and Venereology, Gadjah Mada University Academic Hospital, Yogyakarta, Indonesia; ^11^Department of Dermatology and Venereology, Indonesia University Hospital, Depok, Indonesia; ^12^Department of Dermatology and Venereology, Hasanuddin University Hospital, Makassar, Indonesia; ^13^Department of Dermatology and Venereology, Dr. Syaiful Anwar Regional Hospital, Malang, Indonesia; ^14^Department of Dermatology and Venereology, Dr. M. Djamil General Hospital, Padang, Indonesia

## Abstract

Skin damage among healthcare workers has been reported by many centers around the world. Occupational hand dermatitis is one of the most commonly known occupational skin diseases and a socially significant health issue. The use of gloves is one of the risk factors for the occurrence and/or aggravation of hand dermatitis. This cross-sectional study involved healthcare workers in 14 referral hospitals for COVID-19 throughout Indonesia. Questionnaires were distributed to the participants, which consisted of the subject's characteristics, glove-related skin problems, history of glove use, and clinical history. This study involved a total of 845 healthcare workers. Approximately 156 healthcare workers (18.46%) had glove-induced hand dermatitis during the pandemic. Itchy skin was the most common symptom (44.23%), and the palm was the most frequently complained area (48.72%). There was a significant association between glove use and glove-induced hand dermatitis among healthcare workers. In particular, equal to or more than 2 hours per day of glove use was significantly associated with hand dermatitis. Glove-induced hand dermatitis also had a significant association with the subject's history of atopic dermatitis and previous history of hand dermatitis. The use of gloves by healthcare workers should be considered carefully, especially in individuals at increased risk, including those who use gloves for 2 hours or more per day and those who have a history of atopic or hand dermatitis, in order to prevent the incidence of glove-induced hand dermatitis among healthcare workers, as well as to provide a safe working environment.

## 1. Introduction

Occupational skin disease (OSD) is a skin disorder caused or aggravated by the accumulation of factors and exposure to substances in the work environment [1]. The most common OSD is occupational contact dermatitis (OCD). In general, OCD consists of irritant contact dermatitis (ICD), which is a nonimmunological inflammatory skin reaction that occurs after direct exposure to physical, chemical, and biological substances, while allergic contact dermatitis (ACD) is an immunological inflammatory skin reaction due to contact and penetration of allergens into the skin [2]. Prolonged use of personal protective equipment (PPE) and frequent hand hygiene in the workplace can cause OSD among healthcare workers [3]. After the outbreak of coronavirus disease-19 (COVID-19) and the declaration of the COVID-19 pandemic by WHO in March 2020, intensive transmission prevention measures became mandatory in hospitals and health centers, including the use of PPE and frequent hand hygiene [4]. Skin damage among healthcare workers has been reported by many centers around the world. A study in Hubei, China, found that 97.0% of first-line healthcare workers reported skin damage due to intensive prevention measures against COVID-19 transmission [5]. Meanwhile, a study in Ireland reported that 82.6% of healthcare workers experienced symptoms of dermatitis [6].

Occupational hand dermatitis is one of the most commonly known occupational diseases and a socially significant health issue. Repeated exposure of the skin barrier to irritants is capable of causing damage over time [7]. Hands were one of the three most commonly affected body parts, along with cheeks and the nasal bridge (84.6% hands, 75.4% cheeks, and 71.8% nasal bridge), according to a study in Wuhan, China, about skin reactions among healthcare workers [8]. Hand dermatitis is related to contact allergens and exposure to wet work, particularly rubber additives in medical gloves and fragrances, as well as exposure to soap and water in healthcare settings. Hand dermatitis among healthcare workers during the COVID-19 pandemic has a point prevalence of 14% and a 1-year prevalence of 29%, according to a survey in Sweden [9].

Atopic predisposition, low humidity, frequent hand washing, wet work, the use of occlusive gloves, and the length of working hours are all significant risk factors for the occurrence and/or aggravation of hand dermatitis [10]. People with atopic dermatitis, in particular, already have a disrupted epidermal barrier, which results in higher transepidermal water loss, making them more susceptible to irritants and allergens [2, 11]. Redness, dryness, pruritus, and desquamation of the finger webs or the back of the hand are the first warning symptoms of skin damage, and at this point, a worker must avoid contact with the causative substance or should be relocated for another task [7]. These skin conditions can have a significant negative impact on healthcare workers' morale, capacity to work, and quality of life. Inadvertent PPE violations may result from individuals seeking alleviation from such symptoms, which raises the risk of COVID-19 transmission [12]. This study aimed to evaluate the incidence of glove-induced hand dermatitis among healthcare workers along with possible risk factors to provide a better prevention strategy for the disease.

## 2. Materials and Methods

The population for this cross-sectional study consisted of healthcare workers in 14 referral hospitals for COVID-19 around Indonesia. The inclusion criteria were healthcare workers who were directly or indirectly involved in COVID-19 patient care, had used PPE during the pandemic, and had agreed to participate in the study. Healthcare workers with acute or chronic diseases that might interfere with the study's results were excluded. Total sampling was used as the sampling technique. This study was carried out for six months by distributing questionnaires to healthcare workers. The questionnaire consisted of the subject's characteristics, skin problems related to gloves, history of glove use, frequency of hand washing, and clinical history. The data were collected and sorted according to the purpose of the study, particularly glove use, symptoms of hand dermatitis related to glove use, history of hand dermatitis, and history of atopic dermatitis as possible confounding factors. There is no data loss, and the collected data were analysed for association between variables with Pearson's chi square or Fisher's exact analysis and odds ratio (OR) using SPSS software (Ver. 26; IBM). The ethical clearance of this study was approved by the Ethics Committee of Research in Medical Health, Faculty of Medicine, Public Health, and Nursing, Universitas Gadjah Mada (No. KE/FK/0620/EC).

## 3. Results

This study involved a total of 845 healthcare workers from 14 hospitals in Indonesia. Approximately 156 healthcare workers (18.46%) had glove-induced hand dermatitis during the pandemic. The hospital origin of each participant is summarized in Table S1, and the subject's characteristics can be observed in Table 1. The subjects were predominantly female (81.41%), with 25–29 years old as the largest age group (38.46%). Doctor and nurse were the most prominent professions among the subjects, with 47.43% for each group.

**Figure 1:**
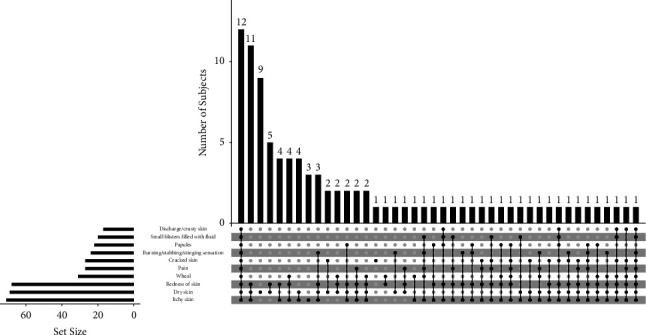
Combinations of symptoms [13, 14].

Table 2 shows that among 156 healthcare workers who had glove-induced hand dermatitis, the most prominent symptoms were itchy skin (44.23%), dry skin (44.23%), and redness of the skin (42.94%). As many as 39.75% of subjects who wear gloves have between 2 and 5 symptoms, the areas most complained about were the palm (48.72%), followed by the back of the hand (288.5%), and the wrist (14.10%). The type of gloves related to the skin complaints was dominated by natural rubber or latex (45.51%), and 20.51% of the subjects had skin complaints after using all types of gloves available in their workplace. The duration of glove use was mostly 4–6 hours a day (28.20%), while the frequency of hand washing was mostly 6–10 times a day (32.05%). More than half of the subjects (57.69%) had never treated their skin complaints, while 35.26% confessed to self-medication. The use of moisturizer was common in 32.05% of the subjects. Approximately 19.23% of the subjects had used topical corticosteroids and 16.67% had used antihistamines to relieve the symptoms they experienced. In this study, one participant may provide multiple responses to the questionnaire. Therefore, a combination of symptoms can be seen in Figure 1.

The clinical history of subjects with glove-related skin complaints is shown in Table 3. There were 15 subjects (9.62%) with a history of hand dermatitis and 12 subjects (7.69%) with a history of atopic dermatitis. A history of allergic contact dermatitis was found in 8.33% of the subjects, while 5.13% of the subjects had a history of irritant contact dermatitis. Approximately 60.26% of the subjects had no history of allergies.

The results of the statistical analyses in Table 4 showed that there was a significant association between glove use in general and glove-induced hand dermatitis among healthcare workers (*p*=0.001). In particular, ≥2 hours per day of glove use was significantly associated with hand dermatitis (*p*=0.018, OR = 1.522, and 95% confidence interval/CI = 1.074–2.157). Glove-induced hand dermatitis also had a significant association with the subject's history of atopic dermatitis (*p*=0.001, OR = 4.018, and 95% CI = 1.820–8.869) and previous history of hand dermatitis (*p* < 0.001, OR = 8.038, and 95% CI = 3.449–18.732).

## 4. Discussion

During the COVID-19 pandemic, gloves, alcohol-based hand rubs, hand washing, and other preventive measures are crucial for preventing the spread of COVID-19. However, frequent hand washing and gloves are also well-known risk factors for the occurrence of hand dermatitis [15]. Hands may be the body part with the most frequent exposure to irritants or allergens and the site of sensitization to allergens in the environment [16]. In order to prevent contact dermatitis and infections, it is important to maintain an intact skin barrier, which is physically located in the stratum corneum [17]. The skin barrier and the skin flora are disrupted by excessive hand washing with soap or alcohol-based products [18, 19]. By disrupting the stratum corneum, soaps, or detergents can make the skin more permeable, susceptible to penetration of irritants/allergens and increase the risk of skin inflammation. Water and soap reduce the natural moisturising component in the skin layers, reducing their ability to bind to water, decreasing skin hydration, and affecting the function of the skin barrier [19].

The participants of this study were dominated by females (67.57%). Doctors (55.15%) and nurses (41.42%) made up the majority of the participants. This study also involved healthcare workers working as laboratory staff, midwives, and other professions, although they only occupied less than 4% of the participants. Survey studies in other countries had similar backgrounds with females as dominant gender, but many studies involved nurses more than doctors [15, 18]. This study revealed that 18.46% (156 out of 845) healthcare workers had self-reported hand dermatitis. The point prevalence of hand dermatitis among healthcare workers, according to a study conducted in Denmark, was 6.4% and 14.9% in another study in Germany [15, 18]. The characteristics of the subjects with hand dermatitis in both studies were similar to those in this study, which was dominated by females. Apart from the fact that 70% of workers in the health and social sectors are women according to WHO, female skin generates less oil that is needed to protect and maintain skin hydration, which puts women at a higher risk of developing contact dermatitis [20, 21].

Various symptoms can be associated with hand dermatitis, including vesiculous and erosion, hyperkeratosis, and desquamation [22]. The most common symptoms found in this study were dry and itchy skin with 44.23% each, followed by redness of skin with 42.94%. One participant may provide multiple responses to the questionnaire, which is a limitation of this study. Self-reported symptoms of hand dermatitis among healthcare workers in Germany showed that dry skin (83.2%), erythema (38.6%), and itching (28.9%) were the most common symptoms [18]. Latex gloves are commonly used by healthcare workers. There have been more and more reports of natural rubber latex hypersensitivity among health care workers, with an incidence of 9.6%. Healthcare workers are more susceptible to developing allergic responses to natural rubber latex. However, all types of gloves have been linked to several adverse skin reactions, such as contact urticaria, ACD, and ICD [23, 24].

Hand dermatitis has many etiologies, such as chemical or physical irritants (in ICD), immediate and delayed hypersensitivity (in ACD), ingested allergens, infection, dyshidrosis, and many others [25]. To identify the cause of hand dermatitis, a thorough history taking, clinical examination of the location and morphology of the lesions, and diagnostic patch tests are recommended in all patients with hand dermatitis with a duration of more than three months and/or relapse [26]. Over 80% of occupational hand dermatitis is caused by ICD, while ACD is the second most frequent type of hand dermatitis [7]. Glove use may affect the temperature, moisture, and pH of the skin, resulting in a disruption of the skin barrier. Frequent glove use combined with exposure to detergents may aggravate the harmful effect of gloves [17].

Among the 156 healthcare workers with glove-induced hand dermatitis in this study, 45.51% confessed that the symptoms they were experiencing started to appear after using latex gloves, followed by all types of gloves (20.51%). Thiurams, rubber accelerator chemicals commonly found in gloves, remain the most common rubber contact allergens found among healthcare workers [27]. Other major glove-related allergies have been reported to carbamates, benzothiazoles, guanidines, and thioureas, among other rubber accelerator chemicals. Vinyl gloves do not include rubber accelerators, but natural rubber latex and nonlatex (such as nitrile) gloves do. Among healthcare workers who underwent patch tests, thiuram mix and carba mix were identified in 8.87 percent and 5.43 percent of subjects, respectively, according to research conducted between 1998 and 2004 [28]. A small number of participants (3.21%) also reported symptoms after the use of prepowdered gloves. Certain powders used in gloves have been linked to an increased risk of skin roughness caused by changes in glove pH. Glove powder has been associated with allergic reactions, and studies have shown that using gloves without powder greatly reduces hand dermatitis [24]. Duration of glove use for 2 hours or more per day and hand dermatitis were found to be significantly associated in this study with OR = 1.522 (95% CI = 1.074–2.157). There was limited investigation of the association between glove use duration and hand dermatitis. Hamnerius et al. found that glove use for >3 hours per day and hand dermatitis were associated with OR = 1.59 (95% CI = 1.36–1.85) [9].

The history of atopy is one of the predisposing factors to hand dermatitis [25]. In patients with atopic dermatitis, the altered skin barrier increases the risk of hapten to penetrate the skin, resulting in an increased risk of developing OCD. Individuals with atopic dermatitis had a three-fold higher probability of developing hand dermatitis, particularly ICD [29]. In this study, it was found that a history of atopic dermatitis and hand dermatitis were associated with glove-induced hand dermatitis. Healthcare workers with a history of atopic dermatitis had a 4-fold higher risk of developing hand dermatitis (OR = 4.018 and 95% CI = 1.820–8.869), and workers with a previous history of hand dermatitis had up to an 8-fold higher risk for hand dermatitis (OR = 8.038 and 95% CI = 3.449–18.732). Another study had reported a lower OR for the association between a history of atopic dermatitis and hand dermatitis (OR = 2.6 and 95% CI = 2.27–2.98), but there was also a study that reported a higher number (OR = 9.04 and 95% CI = 5.21–15.68) [9, 15].

Occupational hand dermatitis is thought of as a minor illness, but if it is not treated, it can develop into a chronic condition that has a big impact on social and professional life. Both preventative actions and therapy are crucial in managing occupational hand dermatitis [7]. All patients should consider making some lifestyle changes. This includes staying away from known allergies and irritants, using alternatives where appropriate, and avoiding wet work and mechanical irritants. Cases involving occupational exposure should be reported to the relevant authority so that the worker can be moved to another task or prevented from coming into contact with the causative substance [7, 26]. Emollients, or moisturizers, are essential in the treatment of all types of dermatitis [22]. Moisturizers should be used regularly and containers should be placed in accessible places around the home and workplace so that they are always within reach. Patients should be informed that some over-the-counter topical products offered by pharmacists, such as moisturizers or antipruritics, can include irritants such as alcohol or propylene glycol [25]. In this study, only 32.05% of subjects with glove-induced hand dermatitis confessed to using moisturizers for their skin complaints. Proper education about disease management should be given to patients with hand dermatitis. It is recommended to give skin protection education and training to high-risk groups such as healthcare workers because it will encourage people to wear proper skin protection and promote a sense of empowerment in terms of taking responsibility for one's own health [26].

## 5. Conclusion

The use of gloves by healthcare workers should be considered carefully, especially in individuals at increased risk, including those who use gloves for 2 hours or more per day and those who have a history of atopic or hand dermatitis, in order to prevent the incidence of glove-induced hand dermatitis among healthcare workers, as well as to provide a safe working environment.

## Figures and Tables

**Table 1:** Characteristics of subjects with glove-induced hand dermatitis.

Characteristics	Total (%)
*Gender*	
Female	127 (81.41)
Male	29 (18.59)
*Age group (years old)*	
20–24	4 (2.56)
25–29	60 (38.46)
30–34	55 (35.26)
35–39	24 (15.38)
40–44	7 (4.48)
45–49	1 (0.64)
50–54	2 (1.28)
≥55	3 (1.92)
*Profession*	
Doctor	74 (47.43)
Nurse	74 (47.43)
Laboratorium staff	4 (2.56)
Midwife	2 (1.28)
Other profession	2 (1.28)
*Working history*	
Less than 1 year	3 (1.92)
1–5 years	65 (41.67)
6–10 years	52 (33.33)
11–15 years	23 (14.74)
16–20 years	6 (3.85)
>20 years	7 (4.49)
*Working hours per week*	
<30 hours	18 (11.54)
30–39 hours	17 (10.90)
40–49 hours	84 (53.85)
≥50 hours	37 (23.72)

**Table 2:** Glove-related skin complaints.

	Total (%)
*Symptom(s) in the last 6 months* ^*∗*^	
Itchy skin	69 (44.23)
Dry skin	69 (44.23)
Redness of skin	67 (42.94)
Wheal	30 (19.23)
Pain	28 (17.94)
Cracked skin	27 (17.30)
Burning/stabbing/stinging sensation	25 (16.02)
Papules	22 (14.10)
Small blisters filled with fluid	20 (12.82)
Discharge/crusty skin	17 (10.89)
*Combinations of symptoms*	
1 symptom	13 (8.34)
>1–<5 symptoms	62 (39.75)
>5 symptoms	18 (11.53)
*Affected area(s) in the last 6 months* ^*∗*^	
Palm	76 (48.72)
1 symptom	11 (7.05)
>1 symptoms	65 (41.67)
Back of the hand	45 (28.85)
1 symptom	7 (4.48)
>1 symptoms	38
Wrist	22 (14.10)
1 symptom	0
>1 symptoms	22 (14.10)
Fingers and between fingers	7 (4.49)
1 symptom	0
>1 symptoms	7 (4.49)
*Type of glove*	
Natural rubber (latex)	71 (45.51)
All types of gloves	32 (20.51)
Synthetic rubber (nitrile, neoprene)	14 (8.98)
Prepowdered rubber gloves	5 (3.21)
Unknown	34 (21.79)
*Duration of glove use per day*	
1-2 hours	29 (18.59)
2–4 hours	35 (22.44)
4–6 hours	44 (28.20)
>6 hours	37 (23.72)
Unknown	11 (7.05)
*Hand-washing frequency*	
0–5 times per day	12 (7.69)
6–10 times per day	50 (32.05)
11–20 times per day	49 (31.41)
>20 times per day	45 (28.85)
*History of medication* ^*∗*^	
Moisturizers	50 (32.05)
Topical corticosteroid	30 (19.23)
Antihistamine	26 (16.67)
Oral corticosteroid	4 (2.56)

^*∗*^One subject might have more than 1 answer.

**Table 3:** Clinical history of subjects with glove-induced hand dermatitis.

Subject's clinical history	Total (%)
Hand dermatitis	15 (9.62)
Allergic rhinitis	15 (9.62)
Allergic contact dermatitis	13 (8.33)
Atopic dermatitis	12 (7.69)
Irritant contact dermatitis	7 (5.13)
Asthma	4 (2.56)
Drug allergy	3 (1.92)
Urticaria	2 (1.28)

^*∗*^One subject might have more than 1 answer.

**Table 4:** Factors affecting glove-induced hand dermatitis among healthcare workers.

	OR (95% CI)	*p* value
Glove-induced hand dermatitis association with history of atopic dermatitis	4.018 (1.820–8.869)	0.001
Glove-induced hand dermatitis association with history of hand dermatitis	8.038 (3.449–18.732)	<0.001
Glove use association with glove-induced hand dermatitis	2.922 (1.536–5.556)	0.001
More than 2 hours per day of glove use association with glove-induced hand dermatitis	1.522 (1.074–2.157)	0.018
More than 4 hours per day of glove use association with glove-induced hand dermatitis	1.683 (1.138–2.489)	0.009

## Data Availability

The descriptive data used to support the findings of this study are restricted by the Ethical Committee of Research in Medical Health, Faculty of Medicine, Public Health, and Nursing, Universitas Gadjah Mada, in order to protect patient privacy. Data are available from Cita Rosita Sigit Prakoeswa, cita-rosita@fk.unair.ac.id, for researchers who meet the criteria for access to confidential data.

## Supplementary Materials

Supplementary Materials Supplementary 1. Table S1: Dr. Sardjito Hospital, Yogyakarta, has the highest percentage of dermatitis-affected healthcare workers, at 16.02%, while Dr. Cipto Mangunkusumo Hospital, Jakarta, has the lowest percentage, at 1.92%.
