# Supplementary material for: Glove-Induced Hand Dermatitis: A Study in Healthcare Workers during COVID-19 Pandemic in Indonesia
Source: Dermatol Res Pract. 2023 Aug 2;2023:6600382. doi: 10.1155/2023/6600382 (PMC10412120; doi:10.1155/2023/6600382)
Supplement: Supplementary Materials — Supplementary 1. Table S1: Dr. Sardjito Hospital, Yogyakarta, has the highest percentage of dermatitis-affected healthcare workers, at 16.02%, while Dr. Cipto Mangunkusumo Hospital, Jakarta, has the lowest percentage, at 1.92%. [file 6600382.f1.docx]

**Table S1.** Healthcare workers from 14 hospitals around Indonesia who had glove-induced hand dermatitis

| **Hospitals** | **Total (%)** |
| --- | --- |
| Dr. Sardjito, Yogyakarta | 25 (16.02) |
| Abdoel Wahab Sjahranie, Samarinda | 16 (10.26) |
| Universitas Hasanudin Makassar | 14 (8.98) |
| Dr. Saiful Anwar Malang | 13 (8.34) |
| Universitas Indonesia, Depok | 12 (7.69) |
| Dr. Hasan Sadikin, Bandung | 12 (7.69) |
| H. Adam Malik, Medan | 11 (7.05) |
| Universitas Gadjah Mada, Yogyakarta | 11 (7.05) |
| Dr. M. Djamil, Padang | 10 (6.41) |
| Dr. Wahidin Sudirohusodo, Makassar | 10 (6.41) |
| Dr. Soetomo, Surabaya | 8 (5.12) |
| Dr. M. Hoesin, Palembang | 7 (4.49) |
| Universitas Sumatera Utara, Medan | 4 (2.57) |
| Dr. Cipto Mangunkusumo, Jakarta | 3 (1.92) |
| Total | 156 |
